# Profile of Class I Histone Deacetylases (HDAC) by Human Dendritic Cells after Alcohol Consumption and *In Vitro* Alcohol Treatment and Their Implication in Oxidative Stress: Role of HDAC Inhibitors Trichostatin A and Mocetinostat

**DOI:** 10.1371/journal.pone.0156421

**Published:** 2016-06-01

**Authors:** Marisela Agudelo, Gloria Figueroa, Tiyash Parira, Adriana Yndart, Karla Muñoz, Venkata Atluri, Thangavel Samikkannu, Madhavan P. Nair

**Affiliations:** 1 Department of Immunology, Herbert Wertheim College of Medicine, Florida International University, Miami, FL, United States of America; 2 Institute on NeuroImmune Pharmacology, Herbert Wertheim College of Medicine, Florida International University, Miami, FL, United States of America; University of Nebraska Medical Center, UNITED STATES

## Abstract

Epigenetic mechanisms have been shown to play a role in alcohol use disorders (AUDs) and may prove to be valuable therapeutic targets. However, the involvement of histone deacetylases (HDACs) on alcohol-induced oxidative stress of human primary monocyte-derived dendritic cells (MDDCs) has not been elucidated. In the current study, we took a novel approach combining *ex vivo*, *in vitro* and *in silico* analyses to elucidate the mechanisms of alcohol-induced oxidative stress and role of HDACs in the periphery. *ex vivo* and *in vitro* analyses of alcohol-modulation of class I HDACs and activity by MDDCs from self-reported alcohol users and non-alcohol users was performed. Additionally, MDDCs treated with alcohol were assessed using qRT-PCR, western blot, and fluorometric assay. The functional effects of alcohol-induce oxidative stress were measured *in vitro* using PCR array and *in silico* using gene expression network analysis. Our findings show, for the first time, that MDDCs from self-reported alcohol users have higher levels of class I HDACs compare to controls and alcohol treatment *in vitro* differentially modulates HDACs expression. Further, HDAC inhibitors (HDACi) blocked alcohol-induction of class I HDACs and modulated alcohol-induced oxidative stress related genes expressed by MDDCs. *In silico* analysis revealed new target genes and pathways on the mode of action of alcohol and HDACi. Findings elucidating the ability of alcohol to modulate class I HDACs may be useful for the treatment of alcohol-induced oxidative damage and may delineate new potential immune-modulatory mechanisms.

## Introduction

Although alcohol consumption has been reported to impair the development of both innate and adaptive immune responses [1], the effects of alcohol on the innate immune system are controversial. Overall, alcohol has been known to be an immune-modulator with high alcohol consumption reported to induce inflammation while moderate alcohol consumption reported to possibly have a beneficial impact on the immune system [2–4]. Dendritic cells (DCs) play a crucial role during inflammation and have been shown to be affected during alcohol abuse. For instance, acute alcohol consumption has been reported to inhibit monocytes and dendritic cell functions [5]. In addition, immune responses induced by chronic alcohol consumption in mice [6] and humans [7] are suggested to be partly due to impaired DC function [8]. Although there is substantial evidence supporting the role of DCs on alcohol-induced inflammation and liver injury [8], the exact mechanisms by which alcohol leads to immune defects are unknown.

One of the mechanisms of interest in this study are histone modifications. Our own previous findings have demonstrated that acute alcohol treatment *in vitro* induced HDAC2 and reactive oxygen species (ROS) in human neuronal cells and these effects were inhibited by trichostatin A (TSA) [9]. Overall, the use of HDACi as potential therapeutic agents to treat alcohol withdrawal symptoms has been reported [10] and chromatin remodeling has been proposed as a novel strategy to control excessive alcohol drinking [11]. Despite recent efforts implicating HDACi in the regulation of transcriptional and behavioral responses caused by several substances of abuse [12–15], studies elucidating the mechanisms of action of alcohol modulation of HDACs and the role TSA and mocetinostat (MGCD0103) play on the alcohol-induced oxidative stress on peripheral cells are scarce. Therefore, in the current study, we present a novel approach combining *ex vivo*, *in vitro*, and *in silico* analyses to elucidate the mechanisms of alcohol-induced oxidative stress and role of HDACs expressed by MDDCs in the peripheral blood.

This is the first study showing evidence of the expression of class I HDACs by monocyte-derived dendritic cells (MDDCs) from individuals that self-reported alcohol use (*ex vivo*) and MDDCs derived from controls treated *in vitro* with alcohol. In addition, this study will present evidence of the ability of alcohol to modulate HDACs enzymatic activity, and the capacity of HDACi to regulate oxidative stress related genes. Moreover, *in silico* analyses based on the *ex vivo* and *in vitro* findings will be presented and may elucidate novel mechanisms of alcohol immune-modulatory effects, interactions of HDACs with oxidative stress responsive genes, potential pathways, and gene-gene interactions.

## Materials and Methods

### Participants

Male and female blood donors were recruited in Miami FL. Written consents were obtained from all the participants prior to the study consistent and approved by Florida International University (FIU) and the National Institutes of Health (NIH) policies. The protocol was approved by the Institutional Review Board (IRB) of FIU, IRB protocol approval # IRB-13-0440. Exclusion criteria for all subjects were polydrug abuse, Hepatitis and HIV, age <18 and >52 years, and pregnancy. Two cohorts were enrolled in the study: controls (*n = 10*) and alcohol users (*n = 10*). Prior to enrollment in the study, all participants completed a questionnaire. Demographics and drinking pattern is provided in S1 Table.

The following cutoff set up by NIAAA guidelines was followed, which considers “at-risk” drinking to be >14 drinks/week (>4 drinks/day) for men and >7 drinks/week (>3 drinks/day) for women [16]. Furthermore, participants that reported to be “at risk” were given the CAGE questionnaire and if two or more responses were answered yes, they were given the Alcohol Use Disorders Identification Test (AUDIT) self- report version developed by the World Health Organization [17]. Although self-report may be considered a limitation of the study, this self-reported screening method is widely supported for the spectrum of alcohol use disorders in various settings with diverse populations [18].

### Differentiation of Cells

For the *ex-vivo* experiments, MDDCs were differentiated from monocytes derived from alcohol users and non-users (controls). For the *in vitro* experiments, MDDCs were differentiated from monocytes derived from controls, then the cells were treated with alcohol. All MDDCs were prepared from peripheral blood mononuclear cells (PBMCs) as previously described by us [19–21]. MDDCs were cultured with complete RPMI media containing 100 U/ml of GM-CSF and 100 U/ml IL-4 (R & D systems, Minneapolis, MN, USA).

### Treatments

Experimental groups include TSA (50–100 nM), MGCD0103 (1, 3, 6 μM), alcohol (20 and 40 mM), alcohol (20 or 40 mM) + TSA (50 nM), alcohol (20 or 40 mM) + MGCD0103 (3 μM). Untreated controls were included and incubated with complete media without any treatments. After allowing the monocytes to differentiate into MDDCs, cells were treated with 0.05% (∼10 mM,), 0.1% (∼20 mM), 0.2% (∼40 mM), 0.3% (~60 mM) and 0.4% (~80 mM) alcohol (catalog # E7023, Sigma Aldrich) for 24 and 48 hrs. These concentrations are equivalent to the physiological blood alcohol concentrations (BAC) of 0.05, 0.1, 0.2, 0.3, and 0.4 g/dL, respectively, and are range below and above the legal limit for driving under intoxication of 0.08% (0.08g/dL). For the HDACi experiments, the cells were treated with 50 and 100 nM of TSA (catalog # T1952, Sigma-Aldrich) or 1, 3, and 6 μM of MGCD0103 (catalog # 726169-73-9, Santa Cruz Biotechnology, Dallas, Texas) prior to alcohol (20–40 mM) treatments.

### Viability Assay

MDDCs were stained with 0.4% trypan blue (Catalog # T8154, Sigma-Aldrich). Live and dead cells were counted using TC20^™^ cell counter (BIO-RAD, Hercules, CA).

### HDACs Gene Expression

For the *ex vivo* experiments, MDDCs were differentiated from human monocytes derived from alcohol users (n = 10) and controls (n = 10). For the *in vitro* experiments, MDDCs were differentiated from human monocytes from controls and were treated with alcohol 0.1 and 0.2% for 24 hours. RNA was extracted and reverse transcribed, followed by qRT- PCR using Taqman assays (Applied Biosystems) for HDAC 1 (Hs02621185_s1), HDAC 2 (Hs00231032_m1), HDAC 3 (Hs00187320_m1), and HDAC 8 (Hs00218503_m1). GAPDH (Hs99999905_m1) and 18sRNA (catalog # 4333760F) were used as internal controls. Gene expression was assessed as previously described by us [9,14,21].

### HDACs Protein Expression

For the protein expression analysis, after MDDCs differentiation, cells were treated with the inhibitors, TSA and MGCD0103 two hours prior to treatment with 0.2% alcohol. MDDCs were harvested and total protein lysates (20 μg) were prepared using the M-PER extraction reagent (catalog # 78501, Thermo Fisher Scientific, Weston, FL). Western blot was performed as previously described by us [9]. Primary antibodies were used against HDAC1, 2, 3 or 8 (Santa Cruz Biotechnology Cat. # sc-81598; Millipore Cat. #04–229; Sigma Aldrich Cat. #H3034; Sigma Aldrich Cat. #H8038) followed by secondary antibody, horseradish peroxidase (HRP)-conjugated anti-goat IgG or anti-mouse IgG (Thermo scientific Cat. # 31402; Sigma Aldrich Cat. # A9044). Same protocol was performed for the *ex vivo* analysis. Total protein lysates (20 μg) from controls (n = 5) and alcohol users (n = 5) were extracted and used. Western blots were analyzed using Image J software. Protein quantification is expressed as % control ± SEM. Optical density (OD) readings were performed at least in duplicates for all samples.

### HDACs Activity

Normal MDDCs were treated for 24 hrs. with 0.1 and 0.2% alcohol (n = 3) followed by NE-PER nuclear and cytoplasmic extraction (Thermo Scientific, Cat. #78835). Whole cell lysate extraction was performed for MDDCs from alcohol users (n = 6) and controls (n = 6). HDAC enzyme activity was measured with the HDAC assay kit following manufacture's protocol (Active Motif, Cat. #102209) and as previously described [14,22]. HDAC enzyme activity was calculated across the groups and graph as pmoles/min/mg of protein. The fluorescence was measured in a Biotek plate reader (Winooski, VT), 360 nm excitation and 460 nm emission.

### Oxidative Stress and Antioxidant Defense Array

Non-treated, pre-treated (50 nM TSA, and/or 3 μM MGCD0103), and/or treated MDDCs (0.2% EtOH) were harvested and mRNA isolation was performed using Illustra triplePrep Kit (GE Healthcare Life Sciences, UK; Cat # 28-9425-44). cDNA synthesis using SABiosciences' RT2 First Strand Kit (Cat # 330401) was performed as per supplier's protocol. Gene profiling was done using RT^2^ profile PCR array kit (SABiosciences, Cat # PAHS-065Z) and assessed with qRT-PCR using Stratagene Mx3000p instrument. Arrays were performed a minimum of two times for each treatment. Functional gene grouping profiles were provided as available online at http://www.sabiosciences.com/rt_pcr_product/HTML/PAHS-065A.html. PCR array data were analyzed using the GeneGlobe Data Analysis Center on QIAGEN’s website. https://www.qiagen.com/us/products/genes%20and%20pathways/data-analysis-center-overview-page/

### *In Silico* Analysis

Target genes identified with the *in vitro* PCR array were further analyzed *in silico* using the GNCPro Gene Network Central research tool (QIAGEN-SABiosciences, Valencia, CA) for collating gene and pathway interactions. Since our study is focus on blood derived immune cells, we proceeded to perform further analysis to predict tissue (whole blood)-specific gene expression associations. Therefore, interactions between HDACs, antioxidant genes, genes involved in superoxide metabolism, and oxidative stress responsive genes were delineated.

### Statistical Data Analysis

Data were analyzed using GraphPad Prism software and microsoft excel. Comparisons between groups were performed using *t*-test or one-way ANOVA and Dunnett’s Multiple Comparison post-test. Differences were considered significant at p ≤ 0.05. Data are expressed as mean ± SE. Experiments were performed at least three times in duplicates or otherwise indicated in the figure legends.

## Results

### Effects of Alcohol or HDACi (TSA and MGCD0103) on MDDCs Viability

**Fig 1:**
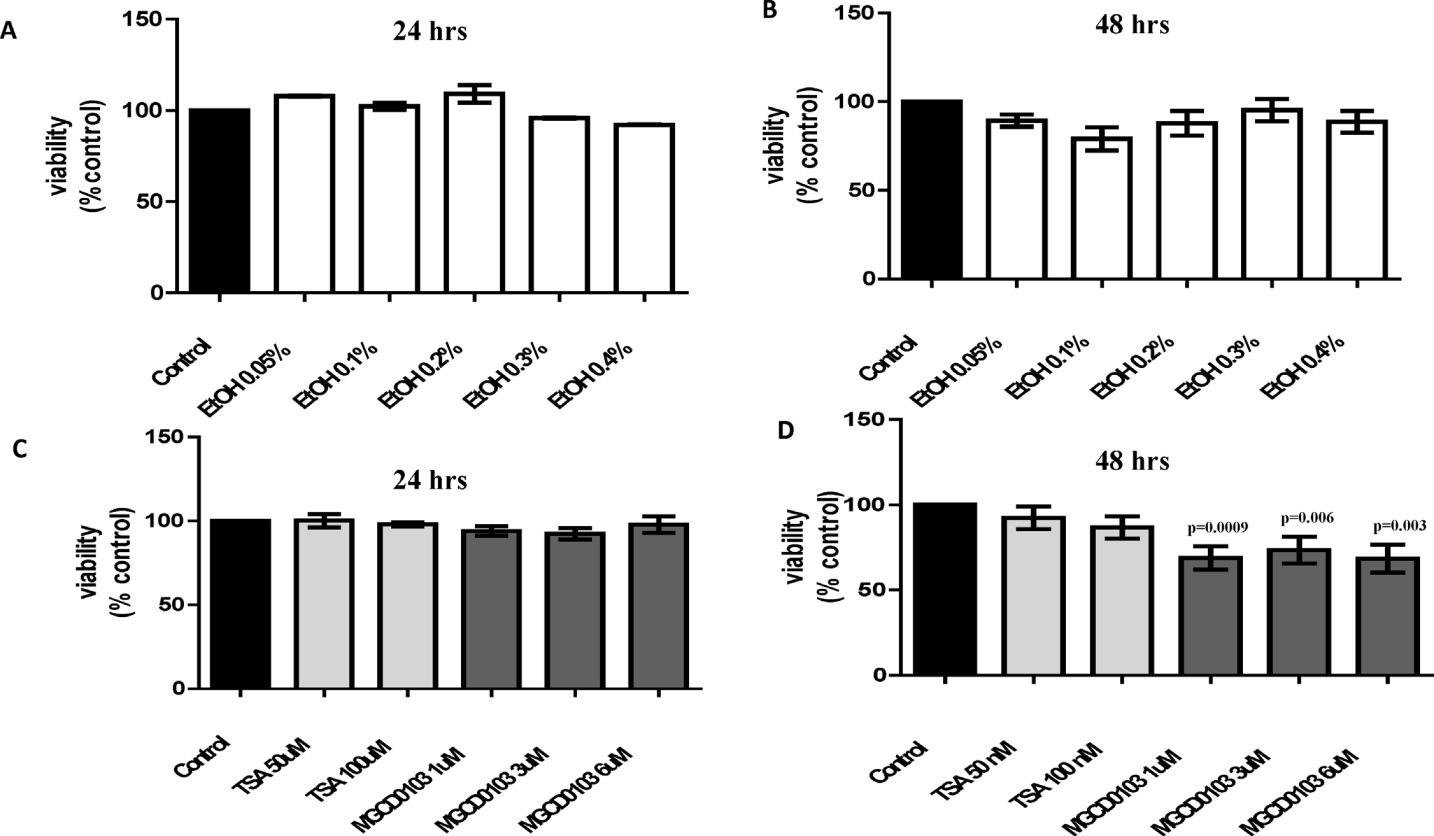
Effects of Alcohol or HDACi (TSA and MGCD0103) on MDDCs Viability. MDDCs were treated for 24 and 48 hours with **A and B**) different percentages of alcohol (EtOH 0.05–0.4%) and **C and D**) different concentrations of HDACi: TSA (50-100nM) and MGCD0103 (1–6μM). Viability was assessed by dye exclusion method using trypan blue. Data are expressed as averages of percent viable cells normalized to control ± SE, (n = 7).

Alcohol (0.05–0.4%) treatments for 24 and 48 hrs. did not have a significant effect on MDDCs viability (Fig 1A and 1B). However, when the cells were treated with the HDACi, TSA and MGCD0103, there was a reduction in viability across the HDACi treated groups compared to the untreated control by 48 hrs. and this reduction was more profound on the MGCD0103 treated cells (Fig 1D). At the 24 hrs time point, HDACi treatments did not affected viability. Based on viability results, our previous reports [9,23], and IC_50_ values reported for single cell viability assays [24,25], the optimum time point (24 hrs) and concentrations of alcohol (0.1 and 0.2%), TSA (50 nM), and MGCD0103 (3 μM) were selected for subsequent studies.

### Alcohol Intake Induces Higher Gene Expression of All Class I HDACs by MDDCs *ex vivo* and after 0.2% Treatment *in vitro*

**Fig 2:**
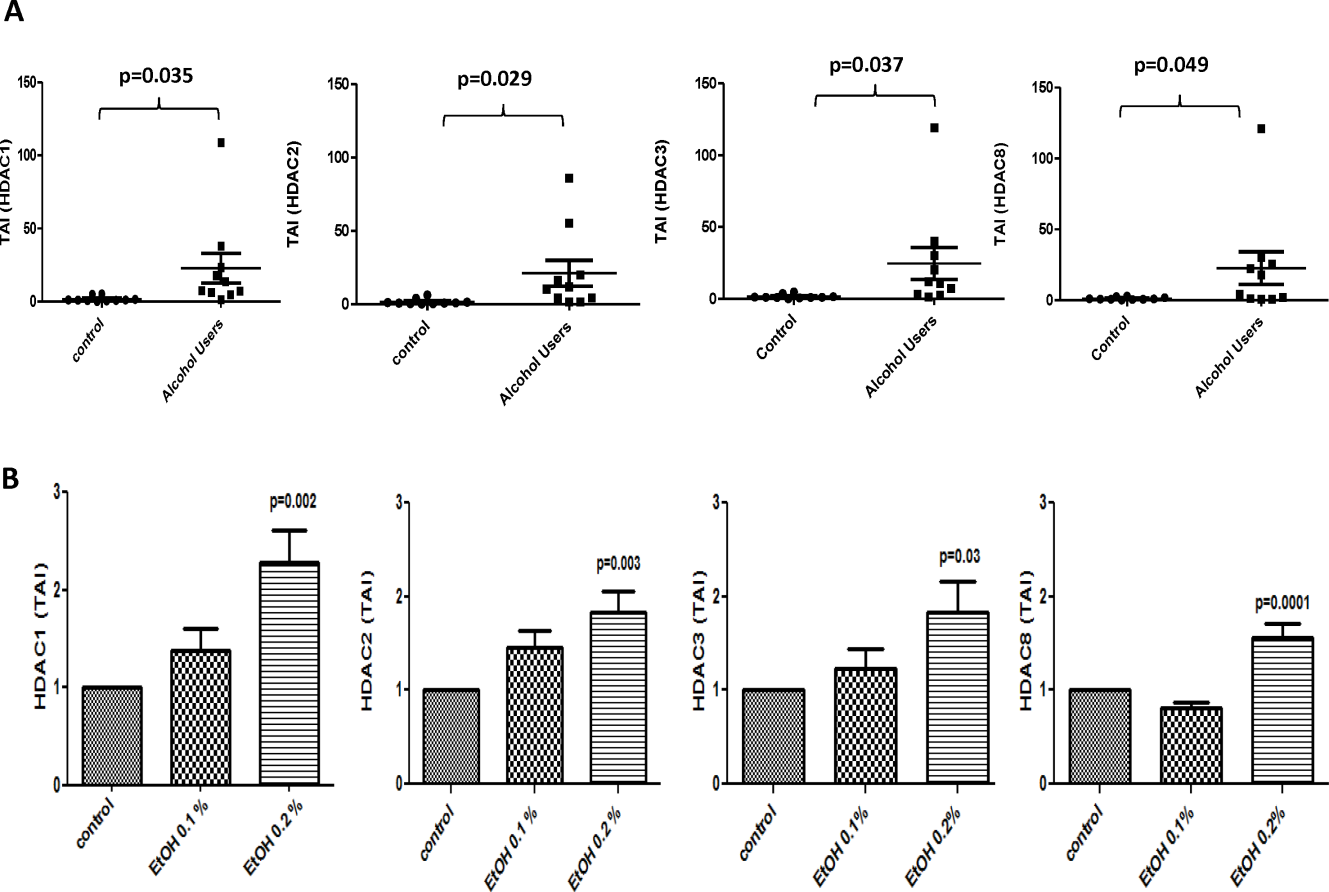
Alcohol Intake Induces Higher Gene Expression of All Class I HDACs by MDDCs *ex vivo* and after 0.2% Treatment *in vitro*. **A)** MDDCs from alcohol users (n = 10) and controls (n = 10) were used to perform gene expression analysis by qRT-PCR. Data are expressed as mean ± SE of TAI values. **B)** normal MDDCs were treated with alcohol, 0.1% (0.1g/dL) and 0.2% (0.2g/dL) EtOH, for 24 hours. Data are expressed as mean ± SE of TAI values of at least three individual experiments performed in triplicates. p≤0.05 is considered significant.

Results in Fig 2A show MDDCs from alcohol users (n = 10) express significantly higher levels of all class I HDACs when compared to the controls (n = 10) as shown by the mean of transcript accumulation index (TAI). HDAC1 [TAI mean (SEM) controls = 1.67 ± 0.58 vs. TAI mean (SEM) alcohol users = 22.79 ± 10.16; p = 0.035]; HDAC2 [TAI mean (SEM) controls = 1.66 ± 0.62 vs. TAI mean (SEM) alcohol users = 21.13 ± 8.78; p = 0.029]; HDAC3 [TAI mean (SEM) controls = 1.60 ± 0.47) vs. TAI mean (SEM) alcohol users = 24.61 ± 11.22; p = 0.037] and HDAC8 [TAI mean (SEM) controls = 1.22 ± 0.30 vs. TAI mean (SEM) alcohol users = 22.59 ± 11.53; p = 0.049]. Fig 2B, show a significantly higher expression of all class I HDACs only after higher dose (0.2%) of alcohol treatment. HDAC1 (control = 1 TAI vs. alcohol 0.2% = 2.3 TAI, p = 0.002); HDAC2 (control = 1TAI vs. alcohol 0.2% = 1.8 TAI, p = 0.003); HDAC3 (control = 1TAI vs. alcohol 0.2% = 1.8 TAI, p = 0.03); HDAC8 (control = 1 TAI vs. alcohol 0.2% = 1.6 TAI, p = 0.0001).

### Alcohol Induces Protein Levels of HDACs *ex vivo* in MDDCs from Alcohol Users

**Fig 3:**
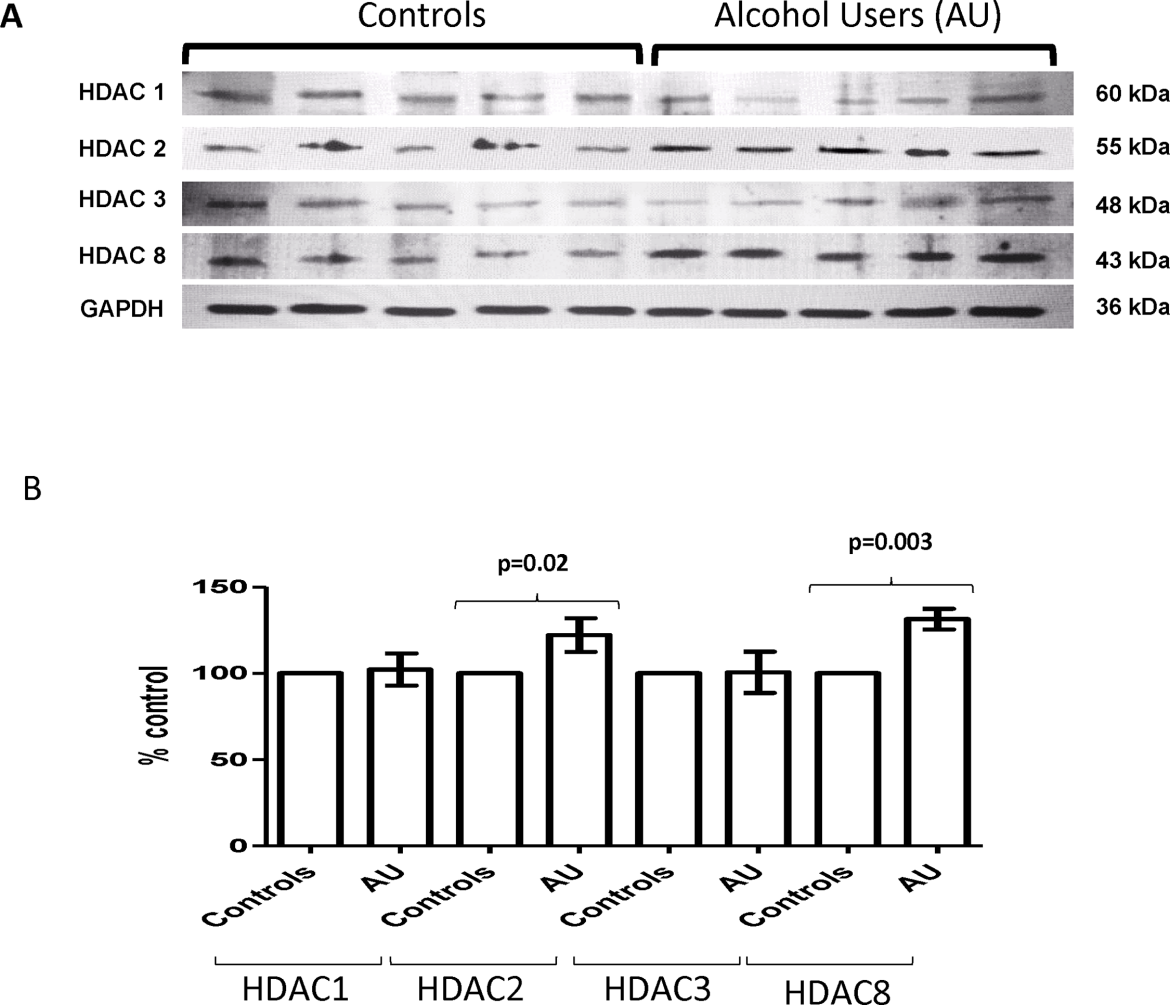
Alcohol Induces Protein Levels of HDACs *ex vivo* in MDDCs from Alcohol Users. **A)** Representative blot is shown for MDDC from alcohol users and non-users. Whole cell lysates (20 μg) were used for western blot analysis. **B)** Optical densities (OD) were analyzed using Image J software. Protein quantification is expressed as percentage of control ± SEM of ten OD readings per HDAC. p≤0.05 is considered significant.

*Ex vivo* results (Fig 3) with MDDCs derived from alcohol users show an increased in protein levels in class I HDACs when compared to controls; however, only protein levels of HDAC2 (mean controls = 100% ± 0 vs. mean alcohol users = 122.2% ± 9.8; p = 0.02) and HDAC8 (mean controls = 100% ± 0 vs. mean alcohol users = 131.4% ± 6; p = 0.0003) were significantly upregulated.

### *In Vitro* HDACi Treatment Significantly Inhibits the Alcohol Effect in all Class I HDACs

**Fig 4:**
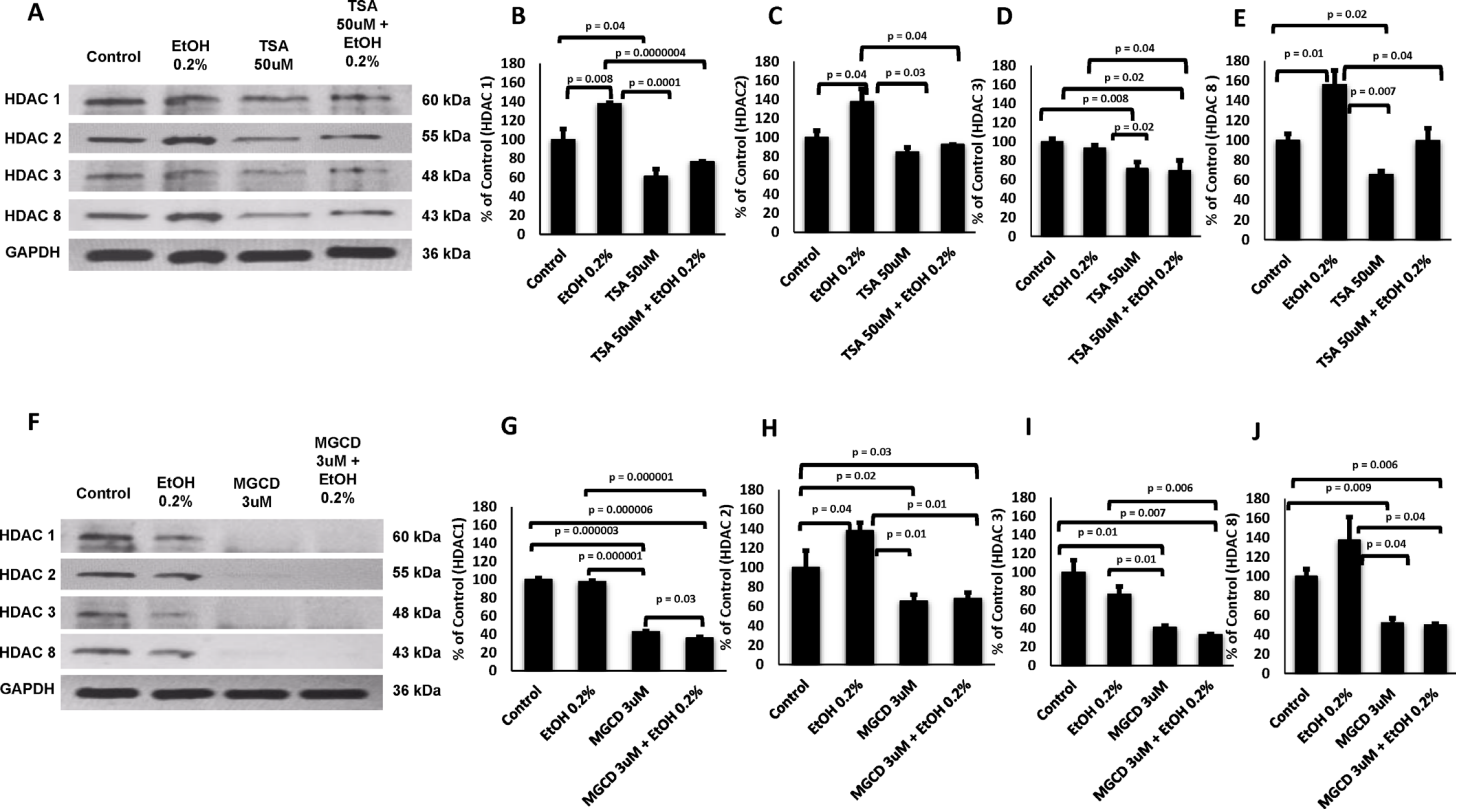
*in vitro* HDACi Treatment Significantly Inhibits the Alcohol Effect in all Class I HDACs. **A and F)** Representative blot is shown for MDDCs pre-treated with TSA (50 nM) or MGCD0103 (3 μM) and/or treated with alcohol (0.2% EtOH, 0.2 g/dL). Total protein lysates (20 μg) were used for western blot analysis. **B-E and G-J)** Protein quantification of two independent experiments. Optical density levels were analyzed using Image J software. Data are expressed as percentage of control ± SEM of two OD readings per HDAC; p≤0.05 is considered significant.

After confirming a major effect of alcohol on the HDACs gene and protein levels ex vivo by MDDCs derived from alcohol users, we proceeded to delineate the specific role of HDACs by pre-treating with the HDACi and then treating with alcohol 0.2% (Fig 4). Our results demonstrated that both HDACi had the capability to cause a visible reduction in all HDACs protein levels as shown by the representative western blot (Fig 4A and 4F); however, the effect of MGCD was more profound. When different experiments were combined to average the optical density values and calculate the percentages of expression with respect to controls, MGCD0103 inhibition of all class I HDACs was significant compared to untreated controls (Fig 4G, 4H, 4I and 4J). Overall, pre-treatment with both HDACi, TSA (Fig 4B, 4C, 4D and 4E) and MGCD (Fig 4G, 4H, 4I and 4J) was able to significantly inhibit the effect of alcohol on all class I HDACs (HDAC 1, HDAC2, HDAC 3 and HDAC8). While pre-treatment with TSA significantly inhibited the effect of alcohol on all HDACs (Fig 4B, 4C, 4D and 4E), TSA alone when compared to untreated control reduced the levels of HDAC2 (Fig 4C); however, this effect was no significant.

### Alcohol Induces HDAC Activity *ex vivo* in MDDCs from Alcohol Users

**Fig 5:**
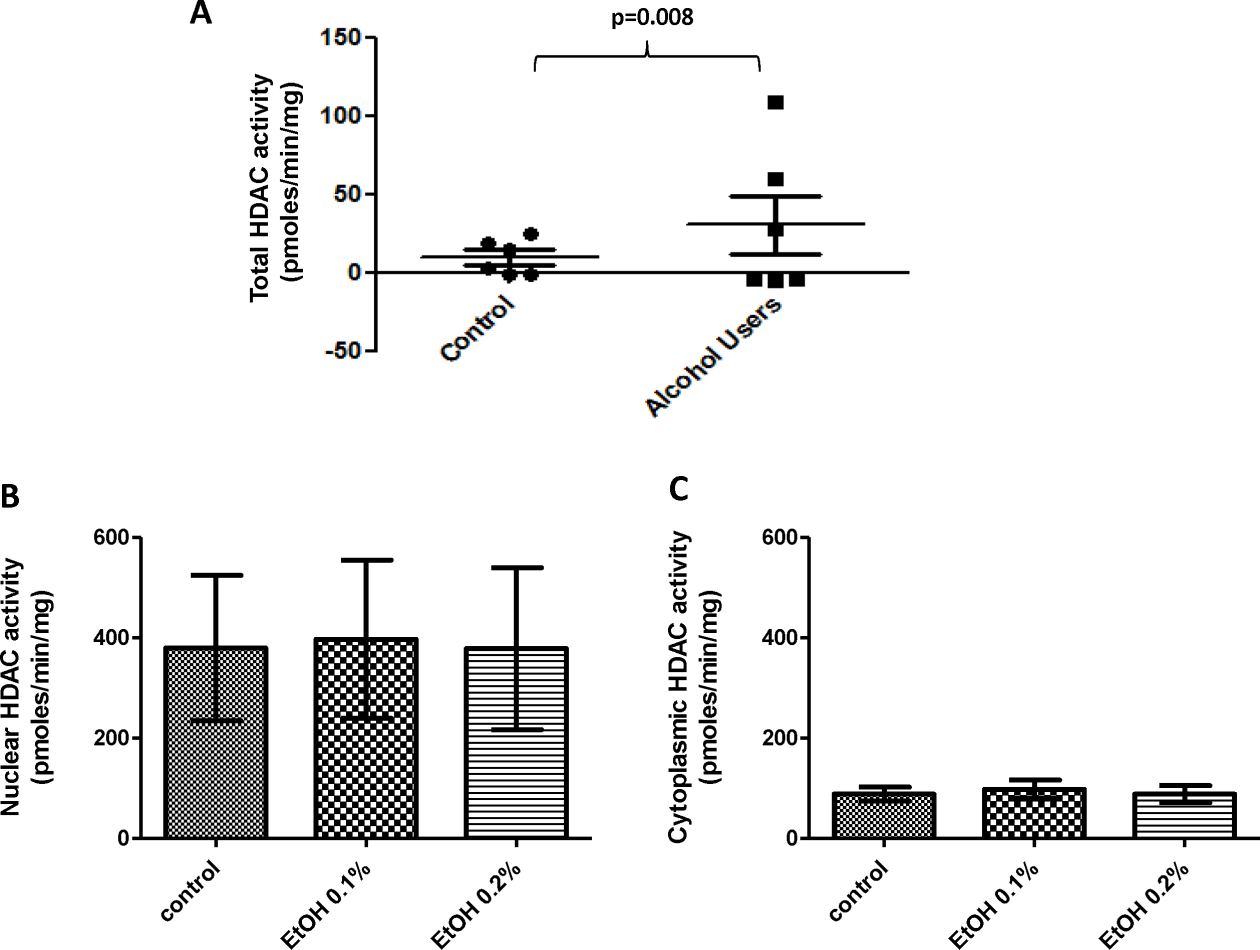
Alcohol Induces HDAC Activity *ex vivo* in MDDCs from Alcohol Users. **A)** Total HDAC activity by MDDCs from alcohol users and controls. **B and C)** Nuclear and Cytoplasmic HDAC activity for alcohol-treated samples (EtOH 0.1% and 0.2%). HDAC enzyme activity was calculated across the groups and graph as pmoles/min/mg of protein. Data are expressed as pmoles/min/mg ± SE of at least three independent experiments. p≤0.05 is considered significant.

We proceeded to test whether the observed increased in HDACs expression was related to changes in HDAC enzymatic activity. Our results (Fig 5A) show a significant increase in HDAC activity *ex vivo* by MDDC from alcohol users compared to controls (controls = 10.2 pmoles/mim/mg vs. alcohol users = 30.8 pmoles/mim/mg; p = 0.008); however, there were no significant differences in activity levels between alcohol and control (Fig 5B and 5C) when the cells were treated *in vitro* with 0.1% and 0.2% alcohol.

### Alcohol Treatment of MDDCs Affects Genes Involved in Antioxidant Response and ROS Production

**Fig 6:**
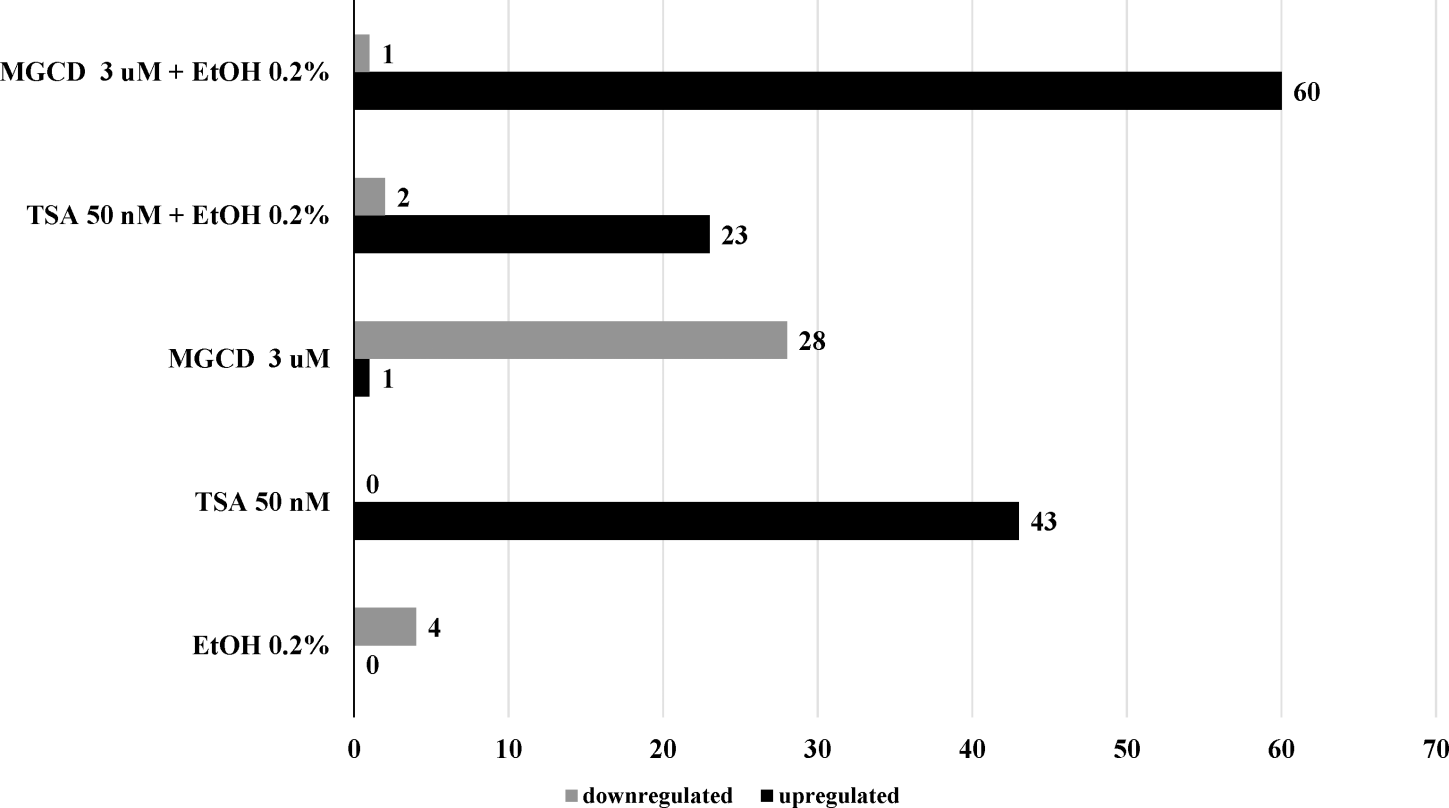
Distribution of Upregulated and Downregulated Genes among Treatment Groups. Out of 84 genes analyzed, only genes (± 5 fold) dysregulated were shown in this graph. Genes were grouped based on their fold upregulation or downregulation after treatment with alcohol (EtOH 0.2%) alone and/ or HDACi, TSA and MGCD0103.

In an effort to identify potential mechanistic targets based on previous reports supporting the ability of alcohol to induce ROS [9,26] and our results highlighting a significant effect of alcohol on HDACs gene expression (Fig 2), we proceeded to explore the functional ability of alcohol 0.2% to modulate genes related to human oxidative stress. Results in Table 1, show that out of 84 genes, 0.2% alcohol treatment had the ability to significantly induce more than five-fold down-regulation of the following genes: CSDE1, CYBA, SGK2, and TXNDC2. TSA and MGCD are inversely regulating oxidative stress since treatment with TSA alone induced upregulation of 43 genes and treatment with MGCD alone induced upregulation of only 1 gene and downregulation of 28 genes. However, when the HDACi where used in combination with alcohol, TSA induced upregulation of 23 genes and downregulation of only 2 genes while the combination of MGCD and alcohol induced the highest upregulation of genes compared with treatment with alcohol or HDACi alone (Fig 6).

**Table 1:** Effects of Alcohol and/or HDACi on Oxidative Stress and Antioxidant Defense Genes.

	EtOH 0.2%	EtOH vs. Control (p value)	TSA 50 nM	MGCD 3 μM	TSA 50 nM + EtOH 0.2%	MGCD 3 μM + EtOH 0.2%
ALB	-1.52	0.349	-2.5	-3.72	**11.72**	**149.25**
ALOX12	-3.25	0.359	**-11.1**	-2.70	1.93	**47.52**
ANGPTL7	-1.63	0.349	-2.89	**13.77**	10.15	**2278.1**
AOX1	1.95	0.596	**-7.38**	-1.54	3.26	**320.59**
APOE	-3.41	0.351	**-12.4**	-12.34	2.56	**15.33**
ATOX1	-2.68	0.355	**-6.16**	-1.45	3.99	**194.23**
BNIP3	-2.93	0.353	**-9.77**	-3.42	2.97	**10.52**
CAT	-2.91	0.352	**-27.4**	**-14.9**	2.31	**20.38**
CCL5	-3.44	0.351	**-8.25**	-3.43	-1.86	**67.16**
CCS	-3.86	0.351	**-6.52**	**-26.18**	-12.41	-1.43
***CSDE1***	**-5.37**	0.02	**-36.9**	**-8.11**	-1.932	**171.2**
***CYBA***	**-5.54**	0.01	-1.54	-1.26	-1.69	**8.892**
CYGB	-4.75	0.335	-3.6	-4.76	**8.21**	**3.965**
DGKK	-1.23	0.459	-3.2	-2.95	**6.80**	**812.15**
DHCR24	-3.87	0.351	-2.02	1.28	3.35	2.473
DUOX1	-2.04	0.348	-3.06	2.49	3.82	**203.6**
DUOX2	-3.66	0.35	-2.61	**-8.27**	2.74	**14.28**
DUSP1	-4.40	0.35	**-6.47**	**-24.42**	1.97	**1910.3**
EPHX2	-2.71	0.352	**-8.11**	-4.84	4.01	1.063
EPX	-2.41	0.357	**-6.51**	-2.31	2.41	**47.39**
FOXM1	-2.99	0.355	**-6.17**	**-9.25**	3.98	**107.6**
GLRX2	-3.78	0.35	**-9.02**	**-25.25**	3.04	**-5.2**
GPR156	-3.17	0.362	-2.22	-1.26	4.18	**2235.8**
GPX1	-4.72	0.35	**-21.7**	-1.17	**-6.38**	**67.91**
GPX2	1.71	0.352	-1.06	1.38	**27.42**	-1.07
GPX3	-1.41	0.349	-2.33	-3.46	**12.60**	**160.41**
GPX4	-1.41	0.349	-2.33	-3.46	**12.60**	**721.87**
GPX5	-2.02	0.359	-2.15	-2.15	5.611	**213.28**
GPX6	-2.19	0.356	**-7.02**	**-6.58**	1.179	**12.26**
GPX7	-2.95	0.354	**-9.76**	**-13.10**	3.00	**28.99**
GSR	-3.14	0.351	**-8.44**	**-11.81**	3.69	-1.05
GSS	-2.73	0.355	**-5.43**	**-17.70**	3.58	**23.32**
GSTZ1	-3.56	0.351	**-8.32**	-4.445	2.544	**69.19**
GTF2I	-3.76	0.352	-4.36	1.086	1.49	**22.07**
KRT1	-4.17	0.349	**-5.57**	-3.350	1.67	-4.40
LPO	-3.53	0.348	**-5.21**	**-33.32**	1.07	**14.99**
MBL2	-2.74	0.349	**-7.57**	-2.92	3.87	**91.05**
MGST3	-1.33	0.367	**-7.57**	-3.06	**9.14**	**92.96**
MPO	-1.61	0.349	-2.66	-3.96	**11.03**	**9868.4**
MPV17	-2.33	0.357	-8.67	**-5.39**	**5.08**	3.524
MSRA	-2.73	0.352	-3.36	**-5.60**	3.38	**62.62**
MT3	-3.15	0.352	-10.8	**-20.88**	3.07	2.64
MTL5	-2.96	0.354	-11.7	**-24.94**	3.02	-1.14
NCF1	-1.41	0.349	-2.33	-1.93	12.60	**5675.8**
NCF2	-3.13	0.35	-2.84	-2.79	2.25	**37.80**
NME5	-2.78	0.351	**-7.05**	**-25.00**	2.85	**-5.14**
NOS2	-2.42	0.349	-3.98	1.10	**7.35**	**93.67**
NOX5	-1.41	0.349	-2.33	-3.46	**12.60**	**524.79**
NUDT1	-1.78	0.349	-3.05	2.15	**9.61**	**1746.9**
OXR1	-3.18	0.353	**-9.73**	**-36.46**	3.75	2.95
OXSR1	-3.38	0.35	**-6.55**	**-10.17**	3.07	**21.31**
PDLIM1	-2.63	0.352	**-7.72**	-2.25	2.87	**56.32**
IPCEF1	-2.25	0.354	**-8.9**	-3.483	4.78	**35.74**
PNKP	-4.04	0.351	**-5.99**	2.66	4.37	**363.7**
PRDX1	-3.82	0.348	**-6.27**	-3.74	1.92	**14.98**
PRDX2	-2.65	0.35	**-9.08**	**-6.90**	3.90	**30.73**
PRDX3	-2.91	0.35	**-8.33**	-1.56	**5.02**	**184.9**
PRDX4	-1.35	0.37	-4.14	-4.48	**7.49**	**20.54**
PRDX5	-2.42	0.361	-2.89	-3.47	-2.39	**80.53**
PRDX6	-3.53	0.35	-7.07	**-6.40**	-3.76	-1.13
PREX1	-3.87	0.271	-2.32	-2.96	**19.78**	**7.43**
PRG3	-3.27	0.35	**-7.92**	-4.38	2.90	**32.50**
PRNP	-2.09	0.363	**-6.32**	**-10.38**	3.76	1.89
PTGS1	-2.41	0.357	**-14.4**	**-55.42**	3.43	1.29
PTGS2	-2.53	0.352	-4.09	2.38	2.58	**53.57**
PXDN	-1.63	0.348	-2.79	-1.09	**10.65**	**3144.5**
PXDNL	-1.87	0.356	**-8.13**	**-15.54**	**6.041**	-4.51
RNF7	-1.58	0.349	-2.61	-3.89	**11.23**	**2682.9**
SCARA3	-2.36	0.352	-5.24	**-9.02**	2.73	4.09
VIMP	-1.74	0.352	-1.01	1.41	4.40	**12.96**
SEPP1	-1.89	0.349	-3.12	-3.06	**9.40**	**3778.5**
SFTPD	-3.22	0.351	**-5.08**	-4.20	-2.71	**9.445**
***SGK2***	**-15.59**	0.34	**-7.71**	**-7.27**	6.56	3.93
SIRT2	-3.10	0.35	-4.52	-1.71	2.71	-118
SOD1	-1.318	0.349	-2.33	-1.20	**12.60**	**17506**
SOD2	-3.01	0.351	**-8.39**	**-8.72**	2.60	4.64
SOD3	-2.02	0.356	**-7.56**	-1.69	3.06	1.05
SRXN1	-2.84	0.349	**-7**	-10.72	2.84	-5.25
STK25	-1.41	0.349	-2.33	-3.4	**12.60**	**501.67**
TPO	-1.56	0.349	-2.99	-1.54	**5.611**	**5699.4**
TTN	-2.12	0.352	**-10.9**	**-8.52**	**5.392**	**19.06**
***TXNDC2***	**-15.1**	0.11	**-10.2**	**-5.34**	1.204	-1.95
TXNRD1	-4.82	0.347	-3.97	**-34.40**	-1.126	**132.11**
TXNRD2	-3.75	0.092	-1.98	-1.46	-1.246	**22.54**
B2M	-3.28	0.593	1.306	1.31	-1.606	1.21

Out of 84 genes analyzed, only genes (± 5 fold) dysregulated were shown in this table. If the oxidative stress genes change is ± 5 fold, the fold change was represented in bold letters. p values are shown for the EtOH treatment with respect to untreated control (n = 5). Genes that were more than five-fold downregulated are represented in bold and italics ***(CSDE1*, *CYBA*, *SGK2*, *and TXNDC2)*.**

### The Effect of Alcohol on Antioxidant Response Genes and Genes Involved in ROS Production is Differentially Modulated by HDACi

**Fig 7:**
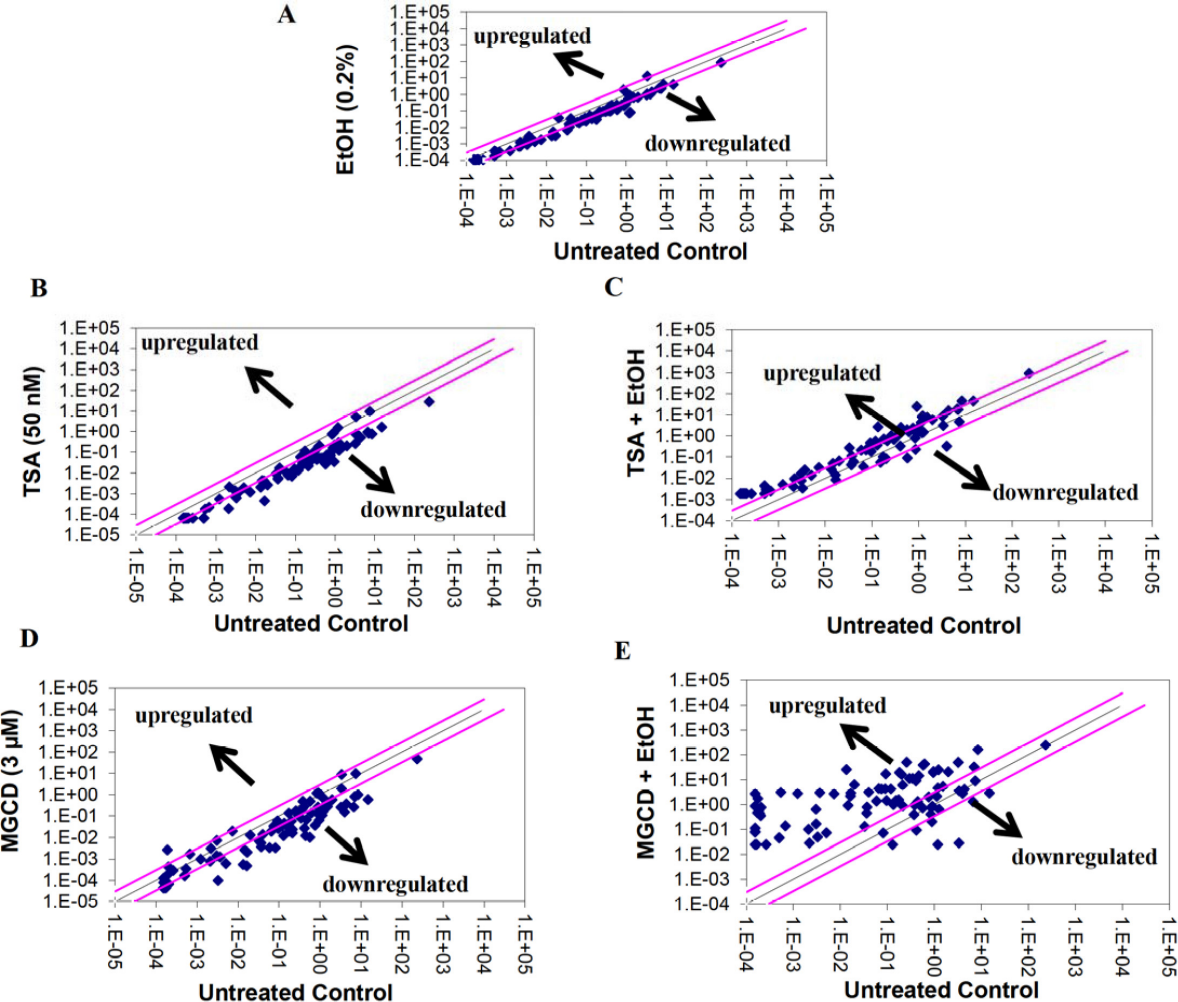
Representative Scatter Plot Analysis of the Changes in Oxidative Stress and Antioxidant Defense Gene Expression by MDDCs Treated with EtOH and/or HDACi. Pair wise comparison of untreated and treated MDDCs with **A)** 0.2% EtOH (0.2g/dL) **B)** TSA (50 nM), **C)** TSA + EtOH, **D)** MGCD0103 (3 μM), and **E)** MGCD0103 + EtOH was performed by scatter plot analysis after running arrays by qRT-PCR. Data were analyzed using GeneGlobe Data Analysis.

**Fig 8:**
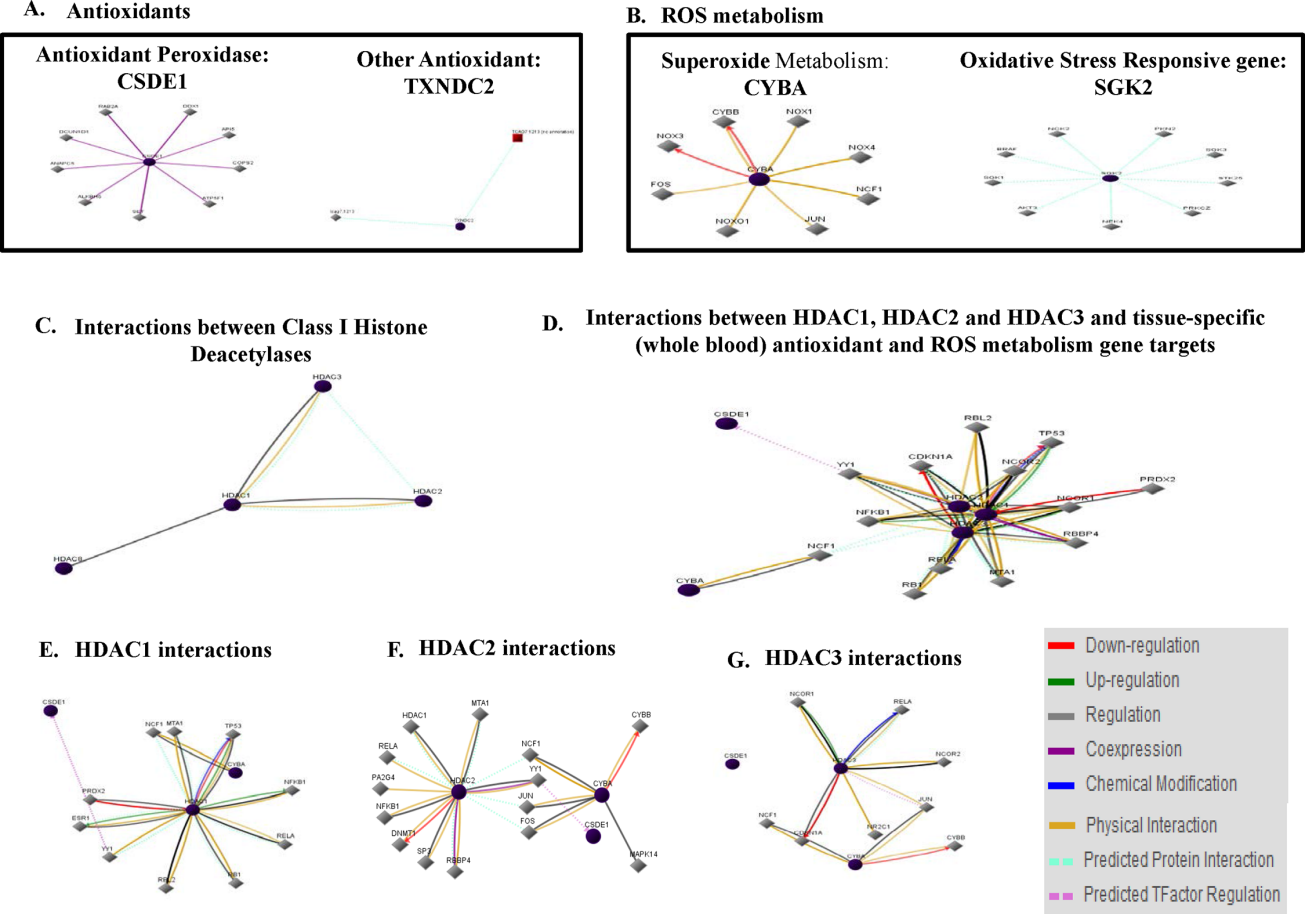
*in silico* Oxidative Stress and Antioxidant Defense Gene Network Interactions with Class I HDACs. Non-specific interactions between **A)** antioxidant, **B)** ROS metabolism genes, **C)** all class I HDACs. Tissue-specific interactions between **D)** HDAC1, HDAC2 and HDAC3 with PCR array gene targets (CSDE1, CYBA, SGK2, TXNDC2) and individual HDACs interactions including **E)** HDAC1, **F)** HDAC2, and **G)** HDAC3. Interactions are color-coded by down-regulation, up-regulation, regulation, co-expression, chemical modification, physical interaction, predicted protein interaction, and predicted TFactor regulation.

We proceeded to test whether the interactions of alcohol with HDACi, TSA and MGCD0103, were further affecting antioxidant responses and ROS metabolism. Fig 7 shows scatter plot analysis of PCR array data after pair wise comparison of control untreated MDDC cells and MDDCs treated with A) alcohol 0.2%, B) TSA 50 nM, C) TSA+ alcohol, D) MGCD0103 3 μM, and E) MGCD0103 + alcohol. Since we were interested not only on the effects of alcohol, but also on the ability of HDACi to modulate alcohol effects, our results in Table 1 show in bold and italics all the genes that were modulated more than five-fold by alcohol 0.2% and further affected by the HDACi. To elucidate the role of the genes being affected by alcohol and/or HDACi, Table 2 provides a summary of all the genes analyzed and their function. Furthermore, target genes were analyzed using the GNCPro Gene Network Central *in silico* research tool (QIAGEN-SABiosciences, Valencia, CA) for collating gene and pathway interactions as displayed in Fig 8.

**Table 2:** Summary Highlighting functional gene grouping of PCR array profiles.

**Antioxidants**			
**Glutathione Peroxidases (GPx)**	**Peroxiredoxins (TPx)**	**Other Peroxidases**	**Other Antioxidants**
GPX1, GPX2, GPX3, GPX4, GPX5, GPX6, GPX7, GSTZ1.	PRDX1, PRDX2, PRDX3, PRDX4, PRDX5, PRDX6.	CAT, CSDE1*, CYGB, DUOX1, DUOX2, EPX, GPR156, IPCEF1, LPO, MGST3, MPO, PTGS1, PTGS2, PXDN, PXDNL, TPO, TTN.	ALB, APOE, GSR, MT3, SELS, SOD1, SOD3, SRXN1, TXNDC2*, TXNRD1, TXNRD2.
**Genes Involved in Reactive Oxygen Species (ROS) Metabolism**			
**Superoxide Dismutases (SOD)**	**Other Genes Involved in Superoxide Metabolism**	**Other Genes Involved in ROS Metabolism**	**Oxidative Stress Responsive Genes**
SOD1, SOD2, SOD3.	ALOX12, CCS, CYBA*, DUOX1, DUOX2, GTF2I, MT3, NCF1, NCF2, NOS2, NOX5, PREX1, PRG3.	AOX1, BNIP3, EPHX2, MPV17, SFTPD.	ANGPTL7, APOE, ATOX1, CAT, CCL5, CSDE1*, CYGB, DGKK, DHCR24, DUOX1, DUOX2, DUSP1, EPX, FOXM1, GLRX2, GPR156, GPX1, GPX2, GPX3, GPX4, GPX5, GPX6, GPX7, GSS, IPCEF1, KRT1, LPO, MBL2, MPO, MSRA, MTL5, NME5, NUDT1, OXR1, OXSR1, PDLIM1, PNKP, PRDX2, PRDX5, PRDX6, PRNP, RNF7, SCARA3, SELS, SEPP1, SGK2*, SIRT2, SOD1, SOD2, SRXN1, STK25, TPO, TTN, TXNRD2.

*Affected genes (highlighted in bold) were classified according to their function as antioxidants, peroxidases, as well as genes involved in ROS metabolism and oxidative stress responses.

### *In Silico* Network Interactions between Genes Involved in Antioxidant Responses and ROS Metabolism

The PCR array results yielded several target genes with more than five-fold modulation after treatment with alcohol and/or TSA, and/or MGCD0103 including CSDE1, CYBA, SGK2, TXNDC2 (Table 1). Therefore, target genes were analyzed using the GNCPro Gene Network Central *in silico* research tool for collating gene and pathway interactions as displayed in Fig 8A and 8B.

### *In Silico* Tissue-specific Gene Network Interactions between HDACs and Genes Involved in Antioxidant Responses and ROS Metabolism

HDACs interactions were also analyzed *in silico*, showing HDAC1 interacts physically with HDAC2 and HDAC3, and further protein interactions between HDAC1, HDAC2, and HDAC3 have been predicted through GNCPro Gene Network Central computational algorithms (Fig 8C). *in silico* analysis yielded regulative interactions between HDAC1 and HDAC8. Since our study is focus on the periphery and blood derived immune cells, we performed further analysis to discover tissue (whole blood)-specific associations. Therefore, additional pathways and associations between the HDACs (1, 2, and 3) and antioxidant genes (CSDE1 and TXNDC2), genes involved in superoxide metabolism (CYBA), and oxidative stress responsive gene (SGK2) were delineated (Fig 8D, 8E, 8F and 8G).

## Discussion

The overall goal of our study is to elucidate the alcohol-induced peripheral mechanisms of action involving modulation of class I histone deacetylases by human dendritic cells. This is the first study to investigate HDAC expression *ex vivo* by MDDCs from self-reported alcohol users and *in vitro* by MDDCs treated with alcohol, and our main findings are highlighted by the ability of alcohol drinking to result in higher gene (Fig 2A) and protein (Fig 3) expression of class I HDACs by MDDCs. These results are in agreement with previous studies supporting an up-regulation of class I HDACs in humans and animals; however, most of the effects of alcohol on HDACs have been reported on the CNS [9,14,27], hepatic cells [28], cardiac endothelial cells [29], or most recently whole blood [30] leaving expression of class I HDACs by MDDC completely unexplored. For instance, we have reported an increase in HDACs 1, 2 and 3 after ethanol treatment of human CNS cells [9,14]. In addition, reports in mice have demonstrated binge alcohol-mediated increase in hepatic HDAC3 [28] and a major role of HDAC2 in the ventral tegmental area during alcohol withdrawal [27]. Most recently, López-Moreno and colleagues reported both binge consumption of alcohol in humans and daily alcohol self-administration in rats increased HDACs gene expression in whole blood [30]; however, they did not analyze cellular specificity, HDAC protein levels, HDAC activity, nor tested the HDAC inhibitors (TSA and MGCD0103) as presented in our current peripheral study with MDDCs.

Our current findings show, for the first time, higher gene expression of all class I HDACs (HDAC1, 2, 3, and 8) by MDDCs derived from self-reported alcohol users compare to controls (Fig 2A). These *ex vivo* findings correlated with the *in vitro* EtOH treatments of MDDCs; however, only the higher dose of alcohol (0.2%) induced a significant up-regulation of gene expression in all class I HDACs (Fig 2B). This could be explained by the differences in dosages since 0.1% (0.1 g/dL) is closer to the physiological limit of intoxication of 0.08% BAC (0.08 g/dL) while the higher dose of 0.2% (0.2 g/dL) alcohol may have an accentuated effect *in vitro* inducing higher levels of modulation and subsequently leading to oxidative stress. Additionally, many factors such as dosage, BAC, and duration of exposure among others, may explain the differential alterations induced by alcohol since exposure to high alcohol percentages can have a more profound effect on immune responses than exposure to low percentages of alcohol as previously described [3,31]. It is also relevant to point out that for the *ex vivo* experiments, we are using cells derived from participants who have self-reported drinking alcohol with an average of 6 ± 1 days/week and 5 ± 2 drinks/day (S1 Table) and blood alcohol levels (BAL) were not measured at the time of blood collection. Although self-report is a limitation of the study, self-report is considered a reliable approach for measuring use of alcohol and other substances since it provides useful estimates of consumption [32]. Recently, new initiates for improving alcohol consumption surveys have been reported and may become useful to complement self-reported data obtained in studies like ours [33].

Although alcohol consumption resulted in higher expression of HDACs *ex vivo* (Figs 2A and 3), only the higher concentration of alcohol (0.2%) significantly induced all HDACs genes *in vitro* (Fig 2B), and when we tested the specificity of the HDACi to block alcohol-induced effects *in vitro*, both HDACi had the ability to significantly inhibit the effects of alcohol on all class I HDACs protein levels with MGCD0103 having a more profound effect (Fig 4), which infers that the oxidative stress effects observed after treatment with alcohol (Fig 7 and Table 1) might be mediated through class I HDACs, specifically HDAC2 and HDAC8, which are the ones significantly upregulated in alcohol users (Fig 3). In addition, this high expression of class I HDACs in alcohol users is correlating with higher levels of HDAC activity *ex vivo* by MDDCs derived from alcohol users compare to MDDCs derived from controls (Fig 5A). However, when total HDAC activity was analyzed after *in vitro* treatment with alcohol, there were no significant differences in HDAC activity and this was also confirmed when specific activity was analyzed more in depth at the nuclear and cytoplasmic levels (Fig 5B). A possible explanation for the differential alcohol responses observed *ex vivo* versus *in vitro* could be attributed to the alcohol metabolites and the differential expression of alcohol metabolism-related genes as previously described in other models [31]. Moreover, the HDACi differential mode of action could be explained by their specificity and concentration since HDACi do not inhibit all HDAC isoforms to the same extent [34]. HDACi are grouped into pan- and class I-specific inhibitors [35], where hydroxamic acids including TSA are considered pan-HDACi targeting class I, II, and IV HDACs [36] while benzamides including MGCD0103 are class I-specific HDACi [37].

Overall, HDACi have been shown to have anti-inflammatory and neuroprotective effects in the brain and these effects seem to extend to other diseases that share mechanisms of oxidative stress, inflammation, and neuronal cell apoptosis [38,39]. However, HDACi have been also known to mediate the induction of apoptosis and autophagy in a variety of cancer cell lines [34]. After confirming the ability of both HDACi to inhibit alcohol-induced effects on class I HDACs (Fig 4), in an effort to elucidate the epigenetic mechanisms of alcohol-induced oxidative stress in the periphery, we proceeded to evaluate the ability of TSA and MGCD0103 to modulate oxidative stress related genes. Out of 84 genes, there were 4 genes highly downregulated (≥5 fold) by alcohol 0.2% while pre-treatment with MGCD0103 was able to have a major reversal of the alcohol effect on antioxidant responses and ROS metabolism genes (Fig 6 and Table 1), possibly through the regulation of all class I HDACs as shown by our current findings.

The *in silico* analyses predicted protein interactions between HDACs (1, 2, 3); however, only physical interactions were shown between HDAC1 and HDAC2/HDAC3. Moreover, regulatory interactions were predicted between HDAC1 and HDAC2/HDAC3/HDAC8. Further *in silico* analysis (Fig 8D, 8E, 8F and 8G) to discover tissue (whole blood)-specific gene expression profiles revealed additional associations between HDACs (1, 2, 3) and CSDE1 (cold shock domain containing E1, RNA-binding), an antioxidant peroxidase involved in ROS metabolism and oxidative stress responses; and between HDACs (1,2,3) and CYBA (cytochrome B-245, alpha polypeptide) a gene involved in superoxide metabolism. Several transcription factors were also found to have physical interactions with HDACs 1, 2, and 3. For instance, NFkB1 was shown to be increased by HDAC1 and to have physical interactions with HDAC2 and HDAC3 while JUN was shown to have interactions with HDAC3 and predicted protein interactions with HDAC1 and HDAC2. Therefore, for future studies, it is worth translating the *in silico* findings to further elucidate the downstream mechanisms of alcohol-induced immune dysregulation resulting in oxidative stress.

## Conclusion

In summary, this study supports the differential involvement of class I HDACs in alcohol-induce oxidative stress by MDDCs. Studying HDAC profiles in cells derived from alcohol users along with *in vitro* effects of alcohol treatment on immune cells may elucidate the mechanisms of alcohol-induce gene dysregulation subsequently leading to oxidative damage. However, additional studies will be required to clarify the specific mechanisms and the downstream gene targets and pathways that mediate the peripheral effects of alcohol.

## Limitations

A major limitation of this study is the alcohol consumption classification, which was assessed by self-report; therefore, it is subject to self-report biases. However, self-report is considered a reliable approach for measuring use of alcohol and other substances since it can provide useful estimates of consumption [32]. Another current limitation is the small sample size; however, the overall lab project calls for additional recruitment of alcohol users and controls, which is currently underway and will be used for continuation of the study.

## Supporting Information

S1 Fig Alcohol Induces Protein Levels of HDACs *ex vivo* in MDDCs from Alcohol Users. (TIFF)

S2 Fig *in vitro* HDACi Treatment Significantly Inhibits the Alcohol Effect in all Class I HDACs. (TIFF)

S1 Table Demographics and Drinking Pattern of Participants. (DOCX)
