# Supplementary figures and images for: Profile of Class I Histone Deacetylases (HDAC) by Human Dendritic Cells after Alcohol Consumption and In Vitro Alcohol Treatment and Their Implication in Oxidative Stress: Role of HDAC Inhibitors Trichostatin A and Mocetinostat
Source: PLoS One. 2016 Jun 1;11(6):e0156421. doi: 10.1371/journal.pone.0156421 (PMC4889108; doi:10.1371/journal.pone.0156421)

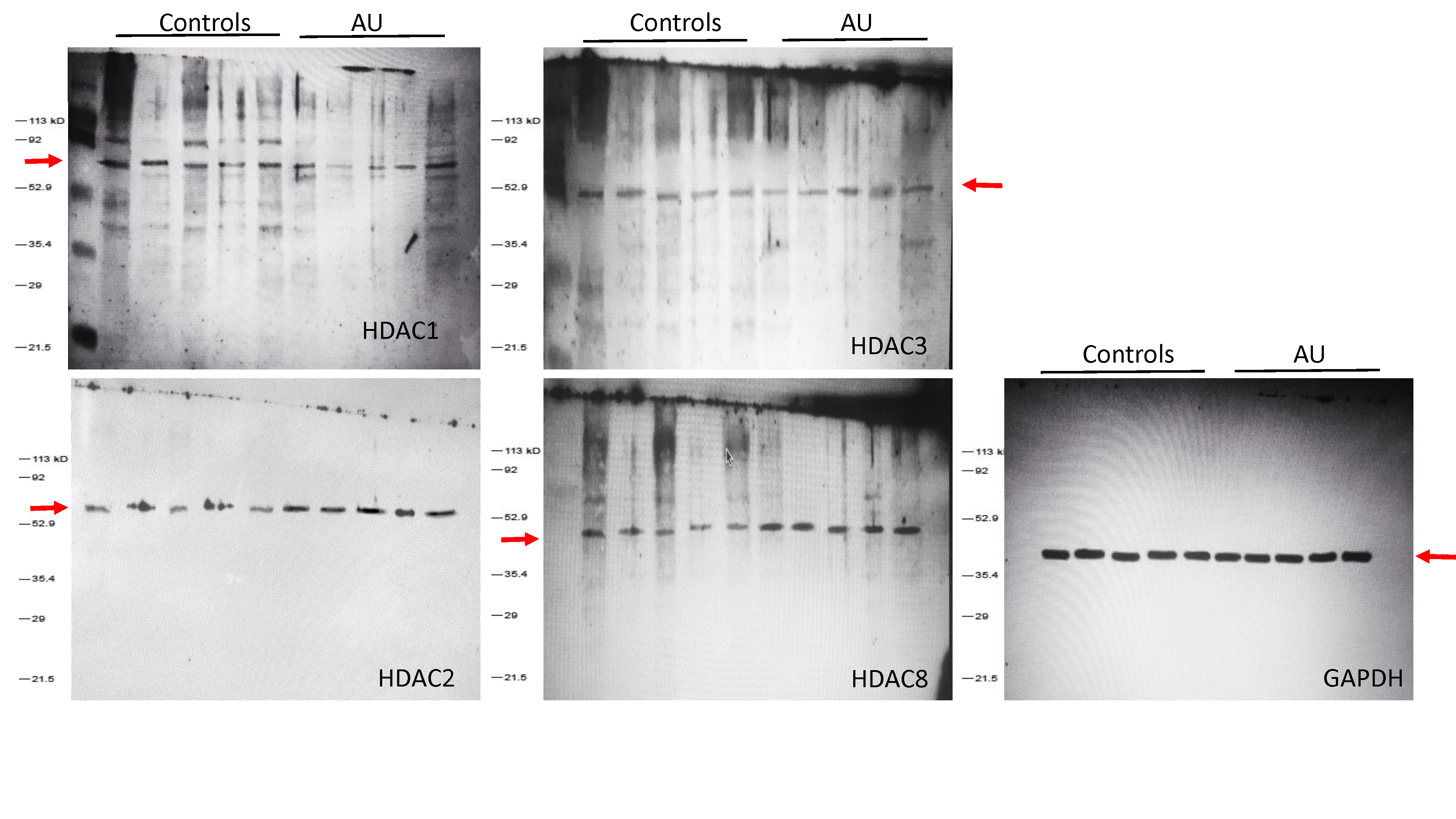

Supplement: S1 Fig — (TIFF) [file pone.0156421.s001.tiff]

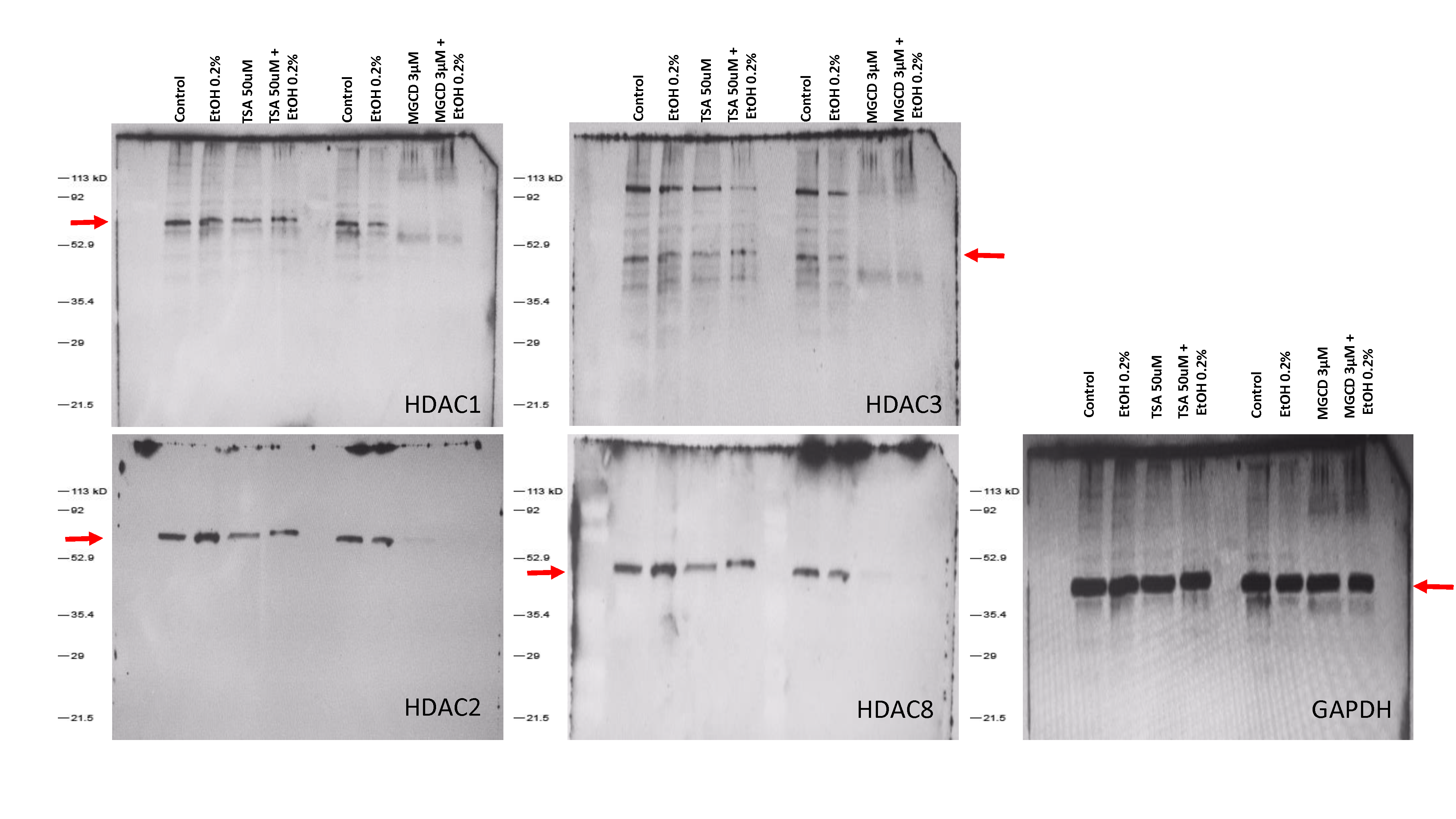

Supplement: S2 Fig — (TIFF) [file pone.0156421.s002.tiff]
