# Supplementary material for: Profile of Class I Histone Deacetylases (HDAC) by Human Dendritic Cells after Alcohol Consumption and In Vitro Alcohol Treatment and Their Implication in Oxidative Stress: Role of HDAC Inhibitors Trichostatin A and Mocetinostat
Source: PLoS One. 2016 Jun 1;11(6):e0156421. doi: 10.1371/journal.pone.0156421 (PMC4889108; doi:10.1371/journal.pone.0156421)
Supplement: S1 Table — (DOCX) [file pone.0156421.s003.docx]

| **Groups** | **Variables** | **All (n=20)** | **Males (n=8)** | **Females (n=12)** |
| --- | --- | --- | --- | --- |
| ***Controls*** | ***Age (mean, SD)*** | 29 ± 9 | 28 ± 3 | 28 ± 10 |
|  | ***Race (%)*** |  |  |  |
|  | African-American | 30% | --- | 43% |
|  | Asian | 20% | 33.3% | 14% |
|  | White | 50% | 66.7% | 43% |
|  | ***Ethnicity*** |  |  |  |
|  | Non-Hispanic | 50% | 33.3% | 57% |
|  | Hispanic | 50% | 66.7% | 43% |
|  | ***Alcohol Drinking (average, SD)*** |  |  |  |
|  | ***Quantity per episode*** |  |  |  |
|  | Drinks/day | 0.3 ± 0.1 | --- | 0.4 ± 0.2 |
|  | Drinks/week | 0.5 ± 0.3 | --- | 0.7 ± 0.4 |
|  | ***Frequency*** |  |  |  |
|  | Drinking days/week | 0.5 ± 0.3 | --- | 0.7 ± 0.4 |
|  | Drinking days/month | 0.8 ± 0.4 | --- | 1.1 ± 0.6 |
|  |  |  |  |  |
| ***Alcohol*** | ***Age (mean, SD)*** | 35 ± 10 | 39 ± 11 | 28 ± 4 |
| ***Users (AU)*** | ***Race (%)*** |  |  |  |
|  | African-American | 10% | 20% | --- |
|  | Asian | 10% | --- | 20% |
|  | White | 80% | 80% | 80% |
|  | ***Ethnicity*** |  |  |  |
|  | Non-Hispanic | 40% | 40% | 40% |
|  | Hispanic | 60% | 60% | 60% |
|  | ***Alcohol Drinking (average, SD)*** |  |  |  |
|  | ***Quantity per episode*** |  |  |  |
|  | Drinks/day | 5 ± 2 | 5 ± 1 | 5 ± 2 |
|  | Drinks/week | 20 ± 5 | 28 ± 10 | 18 ± 0.3 |
|  | ***Frequency*** |  |  |  |
|  | Drinking days/week | 6 ± 1 | 7 ± 1 | 5 ± 2 |
|  | Drinking days/month | 23 ± 5 | 26 ± 4 | 18 ± 7 |
|  |  |  |  |  |

**S1 Table. Demographics and Drinking Pattern of Participants.**
